# Supplementary material for: Qualitative and Quantitative Analysis of C-glycosyl-flavones of Iris lactea Leaves by Liquid Chromatography/Tandem Mass Spectrometry
Source: Molecules. 2018 Dec 18;23(12):3359. doi: 10.3390/molecules23123359 (PMC6320948; doi:10.3390/molecules23123359)
Supplement: Supplementary file 1 [file molecules-23-03359-s001.pdf]

Qualitative and Quantitative Analysis of C-glycosyl-flavones of *Iris lactea* Leaves by Liquid Chromatography/Tandem Mass Spectrometry

Dan Chen [1], Yu Meng[1], Yan Zhu [1], Gang Wu [1], Jun Yuan [2], Minjian Qin [1,*] and Guoyong Xie [1,*]

[1] Department of Resources Science of Traditional Chinese Medicines, School of Traditional Chinese Pharmacy, China Pharmaceutical University, #24 Tongjiaxiang, Gulou District, Nanjing 210009, China. 1721020319@stu.cpu.edu.cn (D.C.); mengyu19900616@163.com (Y.M.); cpuzy@126.com (Y.Z.); woosmail@163.com (G.W.)

[2] Jiangsu Key Laboratory of Regional Resource Exploitation and Medicinal Research, Huaiyin Institute of Technology, Huai'an 223003, Jiangsu, China. yuanjun1109@126.com

* Correspondence: qmj@cpu.edu.cn (M.Q.); 1020142423@cpu.edu.cn (G.X.); Tel.: +86-(0)25-8618-5130 (M.Q. and G.X.)

Supplementary caption

Figure S1. Structure of the twenty-two compounds
Figure S2. Results of single factor experiments
Figure S3. RMS plots for the interaction of the variables in 3D and 2D
Figure S4. Calibration curves of six reference compounds

Table S1. Program and test of RSM
Table S2. Analysis of variance for quadratic model
Table S3. Test result of significance for regression coefficient
Table S4. Results of intra-day precision test
Table S5. Results of inter-day precision test
Table S6. Results of repeatability test
Table S7. Results of stability test
Table S8. Results of recovery test
Table S9. Levels of the response surface test

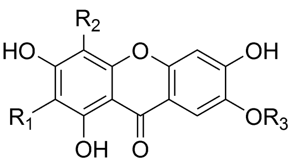

A1: R1=Glc R2=H R3=Glc
A3: R1=Glc R2=H R3=H
A4: R1=H R2=Glc R3=H

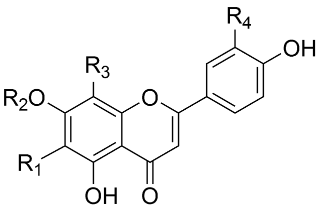

A2: R1=Glc R2=Glc R3=H R4=H
A5: R1=Glc R2=H R3=H R4=OH
A6: R1=Glc R2=CH_3 R3=H R4=OH
A7: R1=Glc R2=H R3=H R4=H
A8: R1=H R2=H R3=Glc R4=OCH_3

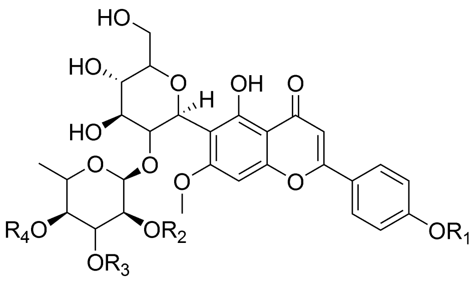

A9: R1=Glc R2=H R3=H R4=H
A10: R1=Glc R2=H R3=H R4=$COCH_3$
A11: R1=CH_3 R2=H R3=H R4=H
A14: R1=CH_3 R2=$COCH_3$ R3=H R4=H
A16: R1=CH_3 R2=H R3=$COCH_3$ R4=H
A18: R1=CH_3 R2=H R3=H R4=$COCH_3$
A21: R1=CH_3 R2=$COCH_3$ R3=$COCH_3$ R4=H
A22: R1=CH_3 R2=$COCH_3$ R3=H R4=$COCH_3$

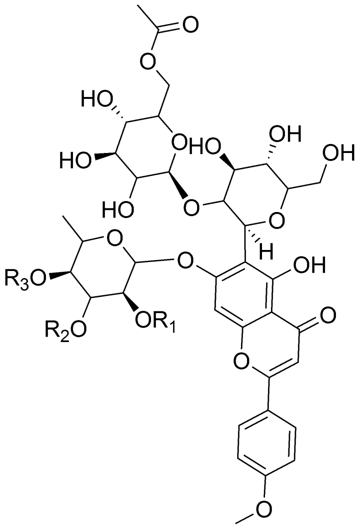

A15: R1=H R2=H R3=$COCH_3$
A19: R1=$COCH_3$ R2=H R3=$COCH_3$

Figure S1

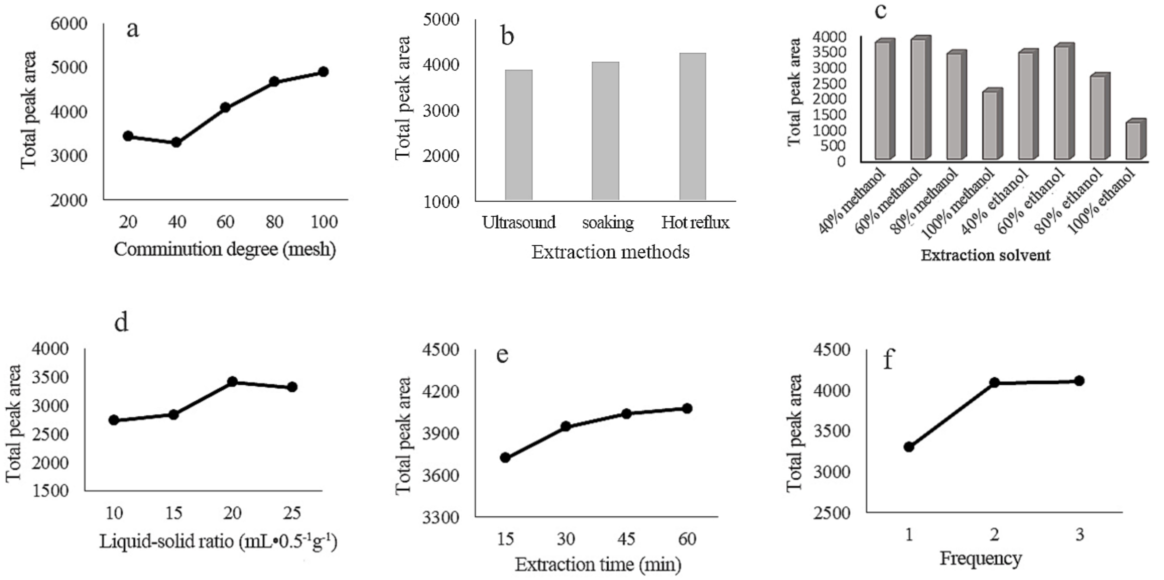

Figure S2

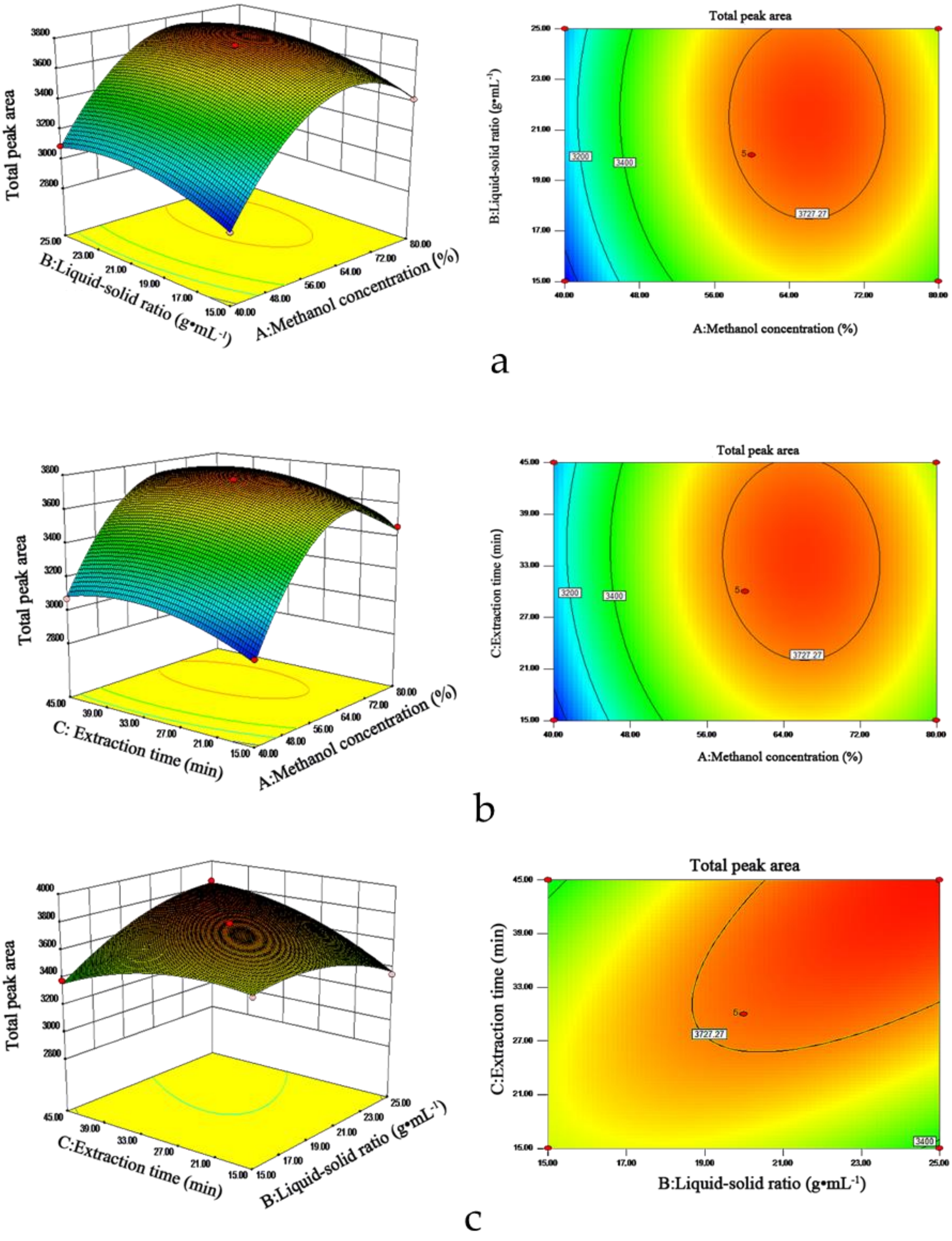
Total peak area
B:Liquid-solid ratio (g•mL⁻¹)
A:Methanol concentration (%)
Total peak area
3200
3400
3727.27
5
a
Total peak area
C: Extraction time (min)
A:Methanol concentration (%)
Total peak area
C:Extraction time (min)
3200
3400
3727.27
5
b
Total peak area
C:Extraction time (min)
B:Liquid-solid ratio (g•mL⁻¹)
Total peak area
3727.27
3400
5
c

Figure S3

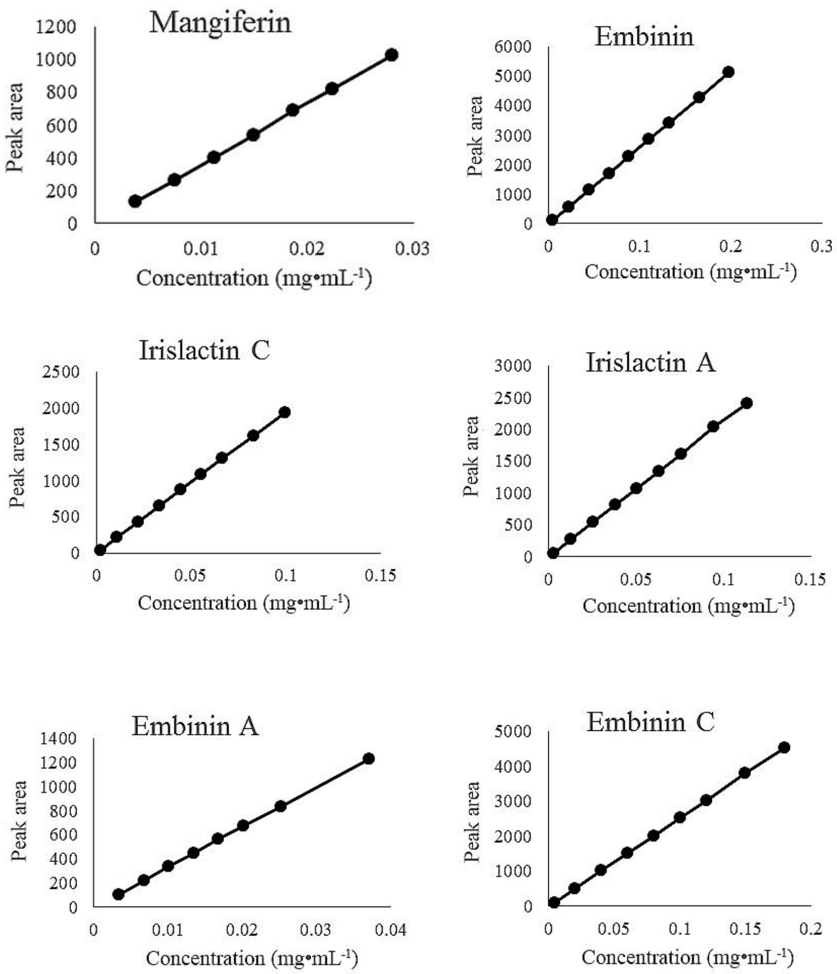
Mangiferin
Peak area
Concentration (mg•mL-1)
Embinin
Peak area
Concentration (mg•mL-1)
Irislactin C
Peak area
Concentration (mg•mL-1)
Irislactin A
Peak area
Concentration (mg•mL-1)
Embinin A
Peak area
Concentration (mg•mL-1)
Embinin C
Peak area
Concentration (mg•mL-1)

Figure S4

Table S1

Std	Run	A (methanol concentration)	B (liquid-solid ratio)	C (extraction time)	Y (total peak area $/\times 10^3$)
**2**	1	80.00	15.00	30.00	3.42
**14**	2	60.00	20.00	30.00	3.76
**8**	3	80.00	20.00	45.00	3.51
**1**	4	40.00	15.00	30.00	2.93
**10**	5	60.00	25.00	15.00	3.35
**15**	6	60.00	20.00	30.00	3.73
**12**	7	60.00	25.00	45.00	3.84
**17**	8	60.00	20.00	30.00	3.75
**4**	9	80.00	25.00	30.00	3.54
**6**	10	80.00	20.00	15.00	3.46
**7**	11	40.00	20.00	45.00	3.07
**3**	12	40.00	25.00	30.00	3.09
**5**	13	40.00	20.00	15.00	2.95
**11**	14	60.00	15.00	45.00	3.38
**13**	15	60.00	20.00	30.00	3.76
**16**	16	60.00	20.00	30.00	3.76
**9**	17	60.00	15.00	15.00	3.56

Table S2

Source	Sum of Squares	df	Mean Square	*F* Value	*P*-Value（Prob>*F*）	
**Model**	1.44	9	0.16	343.38	<0.0001	***
**Residual**	3.255×10^{-3}	7	4.650×10^{-4}			
**Lack of Fit**	2.575×10^{-3}	3	8.535×10^{-4}	5.05	0.0759	
**Pure Error**	6.800×10^{-4}	4	1.700×10^{-4}			
**Cor Total**	1.44	16				
**Adeq Precision**	54.040					
**C.V.%**	0.62					

Table S3

Factor	Coefficient Estimate	Df	Standard Error	95%CI Low	95%CI High	*P*-Value（Prob>*F*）	
**Intercept**	3.75	1	9.644×10^{-3}	3.73	3.77	<0.0001	***
**A**	0.24	1	7.624×10^{-3}	0.22	0.25	<0.0001	***
**B**	0.066	1	7.624×10^{-3}	0.048	0.084	<0.0001	***
**C**	0.060	1	7.624×10^{-3}	0.042	0.078	<0.0001	***
**AB**	-0.010	1	0.011	-0.035	0.015	0.3845	
**AC**	-0.018	1	0.011	-0.043	7.995×10^{-3}	0.1486	
**BC**	0.17	1	0.011	0.14	0.19	<0.0001	***
**A^2**	-0.40	1	0.011	-0.42	-0.37	<0.0001	***
**B^2**	-0.11	1	0.011	-0.14	-0.086	<0.0001	***
**C^2**	-0.11	1	0.011	-0.13	-0.084	<0.0001	***

Table S4

Analytes	1	2	3	4	5	Mean	RSD%
**Mangiferin**	533.9	535.2	535.1	534.9	534.9	534.8	0.10
**Embinin**	2272.5	2279.3	2281.6	2287.3	2291.9	2282.5	0.33
**Irislactin C**	866.3	869.7	871.5	873.4	875.9	871.4	0.42
**Irislactin A**	1068.9	1068.7	1066.8	1064.2	1063.0	1066.3	0.25
**Embinin A**	446.7	447.9	448.7	449.5	450.8	448.7	0.35
**Embinin C**	2013.3	2015.5	2013.6	2012.5	2010.3	2013.0	0.09

Table S5

Analytes	1	2	3	4	5	6	7	8	9	Mean	RSD%
**Mangiferin**	533.9	535.2	535.1	534.9	534.9	535.2	534.8	536.9	540.7	535.7	0.38
**Embinin**	2272.5	2279.3	2281.6	2289.6	2294.3	2300.4	2301.6	2301.8	2305.1	2291.8	0.51
**Irislactin C**	866.3	869.7	871.5	873.2	874.6	877.4	875.9	876.6	878.2	873.7	0.45
**Irislactin A**	1068.9	1068.7	1066.8	1076.2	1074.5	1073.1	1083.4	1076.2	1073.3	1073.5	0.47
**Embinin A**	446.7	447.9	448.7	450.3	451.5	452.8	453.0	453.1	453.9	450.9	0.58
**Embinin C**	2013.3	2015.5	2013.6	2030.4	2033.7	2034.8	2039.6	2037.1	2034.4	2028.0	0.53

Table S6

Analytes	1	2	3	4	5	6	Mean	RSD%
**Mangiferin**	654.8	653.3	659.7	649.9	657.8	659.2	655.8	0.58
**Embinin**	958.8	958.7	967.7	961.5	964.7	973.1	964.1	0.58
**Irislactin C**	623.3	623.7	629.5	631.2	632.8	625.7	627.7	0.64
**Irislactin A**	722.9	723.9	732.5	737.7	733.0	726.4	729.4	0.81
**Embinin A**	1036.2	1035.5	1045.4	1047.7	1049.8	1038.1	1042.1	0.60
**Embinin C**	1593.4	1589.2	1592.2	1594.0	1599.7	1589.7	1593.0	0.24

Table S7

Analytes	0h	12h	24h	36h	48h	60h	72h	Mean	RSD%
**Mangiferin**	468.3	468.6	463.5	470.6	470.2	470.5	472.9	469.2	0.63
**Embinin**	703.9	708.9	711.5	713.7	714.2	717.6	723.8	713.4	0.89
**Irislactin C**	451.3	457.4	454.0	456.5	459.1	462.2	463.1	457.7	0.93
**Irislactin A**	507.2	514.1	516.1	509.8	503.5	507.2	517.8	510.8	1.03
**Embinin A**	762.5	766.5	769.5	766.1	775.7	767.9	777.2	769.3	0.69
**Embinin C**	1154.7	1150.8	1157.8	1162.3	1166.5	1167.6	1167.8	1161.1	0.58

Table S8

Analytes	Sample/mg	Add/mg	Found/mg	Recovery%	Mean%	RSD%
**Mangiferin**	0.451	0.452	0.901	99.72	100.34	1.76
	0.451	0.452	0.892	97.73		
	0.451	0.452	0.907	101.05		
	0.451	0.452	0.913	102.37		
	0.451	0.452	0.911	101.93		
	0.451	0.452	0.899	99.28		
**Embinin**	0.941	0.942	1.889	100.66	99.23	1.83
	0.941	0.942	1.869	98.54		
	0.941	0.942	1.857	97.26		
	0.941	0.942	1.881	99.81		
	0.941	0.942	1.858	97.37		
	0.941	0.942	1.899	101.72		
**Irislactin C**	0.803	0.805	1.613	100.6	99.69	1.97
	0.803	0.805	1.599	98.86		
	0.803	0.805	1.587	97.37		
	0.803	0.805	1.617	101.1		
	0.803	0.805	1.591	97.87		
	0.803	0.805	1.627	102.34		
**Irislactin A**	0.856	0.851	1.688	97.74	100.13	2.14
	0.856	0.851	1.719	101.39		
	0.856	0.851	1.737	103.5		
	0.856	0.851	1.698	98.92		
	0.856	0.851	1.713	100.68		
	0.856	0.851	1.695	98.57		
**Embinin A**	0.781	0.785	1.547	97.54	98.53	1.58
	0.781	0.785	1.572	100.72		
	0.781	0.785	1.551	98.05		
	0.781	0.785	1.568	100.21		
	0.781	0.785	1.542	96.90		
	0.781	0.785	1.549	97.79		
**Embinin C**	1.579	1.575	3.107	96.99	97.62	1.54
	1.579	1.575	3.159	100.29		
	1.579	1.575	3.111	97.24		
	1.579	1.575	3.129	98.39		
	1.579	1.575	3.099	96.48		
	1.579	1.575	3.096	96.29		

Table S9

Levels	Factors		
	A (%)	B (mL 0.5^{-1} g^{-1})	C (min)
-1	40	15:1	15
0	60	20:1	30
1	80	25:1	45
